# Creating Custom Immersive 360-Degree Videos for Use in Clinical and Nonclinical Settings: Tutorial

**DOI:** 10.2196/42154

**Published:** 2023-09-14

**Authors:** Aileen C Naef, Marie-Madlen Jeitziner, Stephan M Jakob, René M Müri, Tobias Nef

**Affiliations:** 1 Gerontechnology and Rehabilitation Group ARTORG Center for Biomedical Engineering Research University of Bern Bern Switzerland; 2 Department of Intensive Care Medicine, Inselspital Bern University Hospital University of Bern Bern Switzerland; 3 Institute of Nursing Science Department of Public Health Faculty of Medicine, University of Basel Basel Switzerland; 4 Department of Neurology, Inselspital Bern University Hospital University of Bern Bern Switzerland

**Keywords:** 360-degree video, head-mounted display, healthcare, relaxing content, technology, video content, video production, virtual reality, VR

## Abstract

The use of virtual reality (VR) stimulation in clinical settings has increased in recent years. In particular, there has been increasing interest in the use of VR stimulation for a variety of purposes, including medical training, pain therapy, and relaxation. Unfortunately, there is still a limited amount of real-world 360-degree content that is both available and suitable for these applications. Therefore, this tutorial paper describes a pipeline for the creation of custom VR content. It covers the planning and designing of content; the selection of appropriate equipment; the creation and processing of footage; and the deployment, visualization, and evaluation of the VR experience. This paper aims to provide a set of guidelines, based on first-hand experience, that readers can use to help create their own 360-degree videos. By discussing and elaborating upon the challenges associated with making 360-degree content, this tutorial can help researchers and health care professionals anticipate and avoid common pitfalls during their own content creation process.

## Introduction

In recent years, there has been an increasing interest in using immersive virtual reality (VR) technology in the clinical setting. This is a continuously growing field, and applications within the clinical setting include, among others, preparedness and medical training for staff, familiarization with the hospital setting, pain treatment, and anxiety treatment [1-8]. Specifically in the intensive care unit, the majority of research done using VR has examined its use with patients as a tool for relaxation [9]. This is followed by its use for delirium prevention in patients; however, the approaches were similar in that they also used VR as a relaxation tool [9].

Due to the increased interest and numerous potential applications for this technology, a group of 21 international VR experts recently worked together to develop a set of standards for best practices [10]. The standards aim to provide guidance when attempting to conduct VR treatments in health care as well as translate findings from VR research into practical applications [10]. The Virtual Reality Clinical Outcomes Research Experts (VR-CORE) committee defined 3 phases that should be used when designing VR clinical studies, starting with content development [10]. The VR-CORE members specifically suggested the use of human-centered design, emphasizing that patients and providers should be involved. This is related to the finding that personalization—allowing the participant to make decisions on various aspects of VR content—can contribute to the level of relaxation and engagement experienced by the user [11]. Furthermore, previous studies have found that the effects of VR across a variety of applications, such as pain therapy and relaxation, are greater when using immersive VR technologies compared with other media such as television screens or headphones [12-16]. Consequently, the application of VR technology ideally requires 360-degree videos that are tailored to their intended purposes.

While specialized companies can be hired to create custom 360-degree videos to suit the specifications of a given project, these concepts and technologies are still relatively new, and their services often come at a premium. Alternatively, immersive content can be purchased on the internet, but this option has its fair share of limitations. While more affordable, users of web-based content must consider the potential licensing issues and royalties associated with its use. Furthermore, videos purchased on the internet may also have limited customizability, such as the length of the content (which is typically restricted to a few minutes), or the location depicted. It may also not be possible to customize certain aspects of the purchased videos, such as the addition of audio tracks (eg, voice-guided meditation) [17,18]. Thus, one potential way of overcoming these limited options is to generate user-created 360-degree video content.

While previous tutorials have outlined how to create 360-degree VR content for training and environmental familiarization, they are limited in their applicability as well as the robustness of the methods described [2,4]. Specifically, these tutorials assume that the creator has access to a controlled environment with minimal risk of interaction with uncontrollable environmental factors. Additionally, these tutorials have failed to address certain steps that are vital for working with 360-degree videos and instead outsourced these steps or used the built-in software provided with the device as a workaround. The limited scope of these existing tutorials, especially in light of the recommendations of the VR-CORE group, highlights a gap in the literature regarding the creation of customizable in-house VR content that does not require the user to outsource certain aspects of the work or hire expensive companies.

The goal of this tutorial paper is to provide readers, be they researchers or health care professionals interested in applying VR in a clinical setting, with a pipeline that can be used to create custom 360-degree videos. Moreover, the goal is that the pipeline can be used for a variety of applications across various levels of expertise and multiple target populations. The methods and advice provided in this paper are based on the study team’s first-hand experience of creating 30-minute, 360-degree nature videos that were subsequently shown to patients with critical illness in an intensive care unit using a head-mounted display (HMD). While the focus of our work was the creation of relaxing scenes that featured nature, the steps outlined below can be easily generalized and used to record content that is more suitable for different purposes, such as pain therapy or distraction, in which the 360-degree exploration of a given setting is desired [5,6,8,19-23].

## Editing Pipeline

### Overview

**Figure 1:**
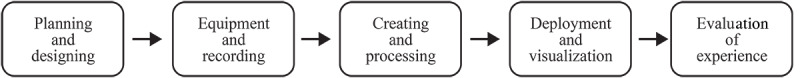
Proposed pipeline for creating and using 360-degree videos.

The pipeline presented in this paper focuses on 5 main aspects that must be considered when creating 360-degree videos (Figure 1).

The first aspect is the planning and designing of the content, which requires making decisions on a variety of parameters, such as the duration of the content, as well as its visual and auditory components. The second aspect covers the auditory and visual equipment necessary as well as how to record the footage. The third aspect involves the creation and processing of the final footage, with a specific focus on how to combine the recorded content into a single 360-degree video and postprocessing considerations. The fourth phase discusses the hardware that should be used for deployment and visualization; it includes considerations regarding the visualization of the 360-degree content, the choice of VR device, and hygiene precautions. Finally, this tutorial discusses how to evaluate the VR experience. Additional detailed descriptions of the experimental setup as well as potential use cases following this pipeline can be found in Multimedia Appendix 1.

### Planning and Designing of the Content

#### Overview

Before beginning to record any content, it is important to know what types of scenes should be recorded and their duration; this will depend on the overall purpose of the VR content. For example, if planning to use VR for relaxation purposes in patients with critical illness, the literature suggests that the duration should ideally be around 10-15 minutes [24]. On the other hand, if VR is to be used as a meditation tool to improve sleep in patients with critical illness, then a duration of 30 minutes may be better suited to that purpose [17]. Relevant points to consider are the visual content, the auditory content, and the duration of the content.

#### Visual Content

The types of scenes to be recorded will depend on the purpose of the content. Specifically, if the content is intended to provide a distraction, then engaging content such as cartoons or interactive scenes, as have been used for pain therapy, may be the best choice of content [12,13,22]. If, however, someone wants to be relaxed, a calm nature scene may be more suitable than an urban environment [19,20,25]. Based on this, Table 1 outlines several important questions that should be considered when deciding what type of content should be recorded.

**Table 1:** Questions and considerations related to the visual content of the videos.

Questions	Considerations
What type of activity should be present? (eg, human activity, animal activity, nonhuman activity)	Some activity should be present, or the video may look like a still image. The type of activity will depend on the individual goals of the VR^a^ stimulation (eg, distraction and relaxation). For example, natural settings are more relaxing than urban settings, so in this case, limit human activity. For scenes involving human activity, local filming laws should be consulted.
How much activity should be present? (eg, constant activity, intermittent activity)	Too much activity, and the viewer can become overwhelmed. Too little activity, and the viewer can become bored [26]. The ideal amount of activity depends on the target population and the goals of the content.
How quickly should the activity be occurring or should the scene be changing?	Content that is too repetitive or does not change enough may be boring. Content that changes too much or too quickly can be confusing or disorienting.
Should the recording be static or dynamic?	Dynamic recordings may be more engaging. Dynamic recordings may increase the risk of cybersickness [27].
What about cybersickness?	There are 5 main contributors to cybersickness: content, interaction, human, hardware, and experimental factors [27]. Some risk factors cannot be controlled (eg, age), while other factors can be accounted for (eg, static versus dynamic content).

^a^VR: virtual reality.

#### Auditory Content

Like visual content, the type of auditory content that should be included is dependent on the goals of the video (eg, voice-guided meditation or relaxing nature sounds) [17,18,28]. Table 2 outlines several important questions that should be considered when recording and postprocessing 360-degree content.

**Table 2:** Questions and considerations related to the auditory content of the videos.

Questions	Considerations
What type of auditory content should be presented?	The addition of music can be challenging as it is very personal [24]. Sounds recorded together with the visual content are both easier to include and will more reliably engage the user [14,24]. Audiobooks or podcasts could be used depending on the goal of the stimulation (eg, nature scene with guided meditation).
Should the auditory input be monophonic or stereophonic?	Stereophonic audio creates a multidirectional perspective on the horizontal or vertical plane (eg, birdsong coming from the left or right). This may depend on the recording equipment available (eg, a built-in camera microphone may restrict the user to monophonic). Spatial audio creates sounds in a 3D space, with the listener in the middle. Spatial audio can be programmed to respond to head movements.
At what volume should the auditory input be presented?	Volume should be regulated throughout and across content. Individuals may have different listening preferences or be hard of hearing. It may not always be feasible for the user to adjust the volume level themselves.

#### Duration of the Content

A final component to consider is the length of the content as well as how it relates to both the visual and auditory content. The ideal video duration is dependent on how the final video will be used as well as the content itself. For example, if the goal is to distract people, as is often the goal in pain therapy [23], then individual videos could be shorter. In contrast, if the goal of the video is to induce a relaxation effect—for example, by showing them a sunset—then the video should be long enough to capture this event. In general, the overall length of the video can be adjusted by adding or removing video footage. Alternatively, the length can be adjusted by ensuring that it can be looped, that is, the end of the video can seamlessly transition into the start of the video without a perceptible difference in the content. In this way, the duration of the footage can be adapted at a later stage (eg, during postprocessing), adding another layer of personalization.

### Equipment and Recording of the VR Content

#### Visual Equipment

As the technology available for recording 360-degree videos continues to evolve, there are an increasing number of commercially available cameras. There are 2 main types of cameras: monoscopic and stereoscopic cameras. Monoscopic VR uses multiple monoscopic cameras attached to a rig to film multiple fields of view that are stitched together in the postprocessing stage. Stereoscopic VR also uses multiple cameras to film multiple fields of view, with the exception being that, in stereoscopic cameras, there is a lens assigned to each eye. In this way, stereoscopic cameras can generate 3D content that cannot be achieved using a monoscopic rig unless special postprocessing techniques are used.

In addition to the camera itself, there are additional accessories that are required. Videos recorded using 360-degree cameras can be extremely large depending on the resolution, frame rate, and duration of the recording and may require additional storage solutions as well as a powerful computer for processing. The exact specifications will depend on the camera used and the video files generated; a more detailed example can be found in Multimedia Appendix 1. The camera may also require supplementary batteries or an additional power source if outdoor scenes are being captured. The environmental conditions may also necessitate the purchase of additional accessories related to wind or rain protection.

**Figure 2:**
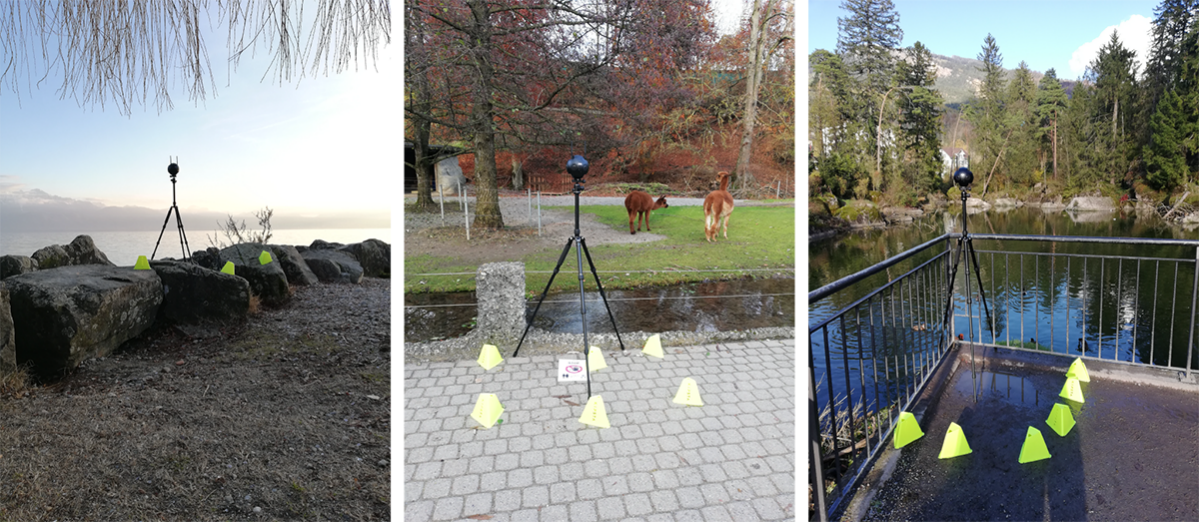
Examples of on-location shooting using fluorescent cones to make passersby aware of the tripod and camera.

Finally, as this setup may appear intriguing to passing individuals, it may be useful to consider some protective measures. Specifically, it may be useful to attach signs to the camera tripod, warning individuals to refrain from approaching or touching the camera, as this may disrupt the recording and have a disorienting effect on the viewer. Florescent cones can also be placed around the base of the tripod to prevent individuals from approaching the camera or accidentally knocking the camera down (Figure 2). Any person or object that comes close to the camera can cause discomfort for the end user, as they might feel that the object approached too close to them. Lastly, as the camera captures footage of its entire surroundings, there is no way for someone who does not wish to be filmed to pass by undetected. To prevent problems associated with this issue, it can be useful to place signs at an appropriate distance from the camera that warn passersby that they will be filmed if they continue on this path. Individuals who do not wish to be filmed can then choose an alternative route.

#### Recording Perspective

**Figure 3:**
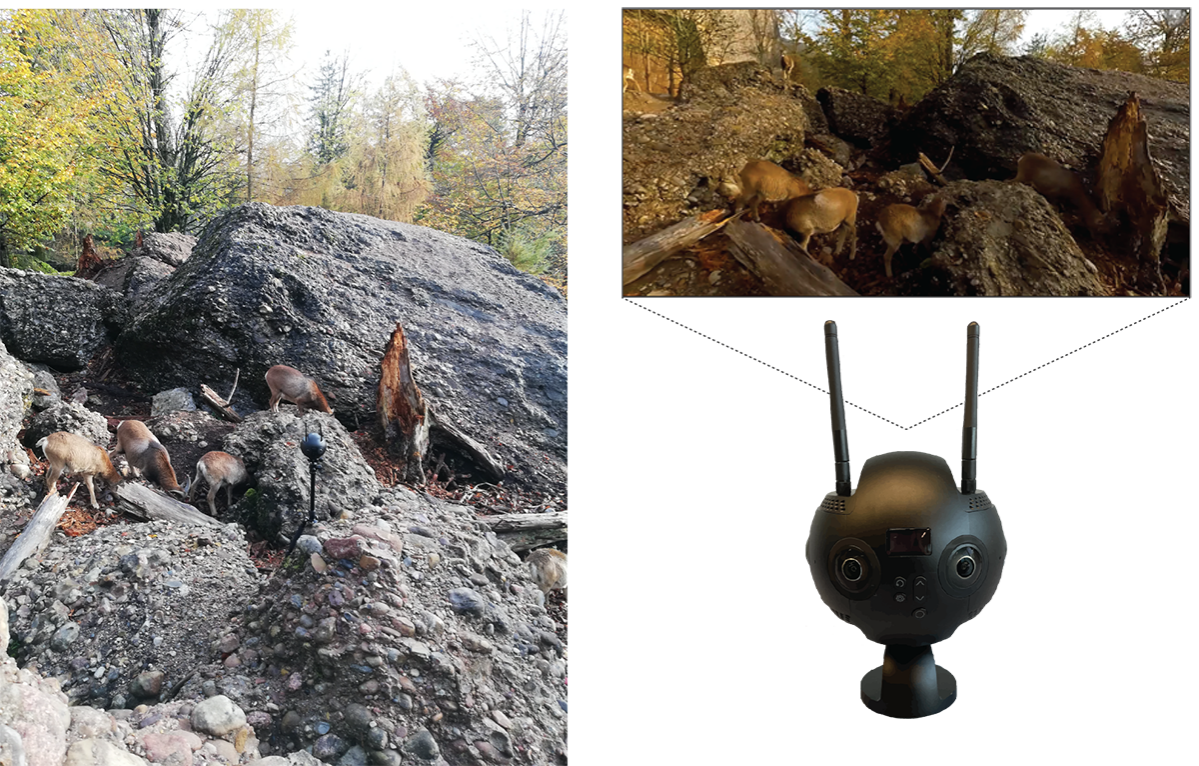
Camera setup and examples. (Left) Camera setup while filming on-location. (Top right) Screenshot of the final postprocessed video as it would be seen using the VR headset. (Bottom right) Insta360 Pro II camera (Arashi Vision Inc) showing the control panel. VR: virtual reality.

When filming on location, the easiest and most flexible way of positioning the camera to capture scenes is through the use of a camera tripod. There are two recommended heights that should be used to guarantee a natural viewing experience: a height that is comparable to someone who is standing or a height that is comparable to someone who is seated on the ground. Selecting 1 of these 2 heights will increase the likelihood that the user will experience the scenes as if they were truly present at that location (Figure 3).

#### Audio Equipment

There are 4 main methods by which auditory content can be added to 360-degree videos. First, one may choose to film the visual content using devices that have built-in microphones. This option allows for the most seamless combination of visual and auditory content. However, recording the auditory content may not be straightforward, depending on the equipment available and used. For example, cameras that have a built-in cooling fan may result in poor-quality audio recordings. Additionally, it is impossible to customize microphones that are built into the camera; users may wish to use an external microphone to record the soundscape.

There are many different devices available depending on budget and the desired specifications, which will be dependent on the content considerations discussed above. Care must be taken to find a recording device with a suitable range that is capable of picking up on activities in close proximity but not extraneous sounds, such as sounds from a distant highway [29]. Additional characteristics that should be considered include the power supply (ie, cabled or battery-powered), internal storage capabilities, and wind protection. The latter is particularly important for outdoor recordings, as even the slightest gust of wind can be heard on audio recordings [29]. If the video content is expected to include a vocal track—for example, to allow for a guided meditation routine or to act out a scene—it may be important to consider how this voice will be captured by the device. A better solution may be to record these audio tracks separately and add them to the video during postprocessing.

In cases where it is not possible to record sounds on location, sound clips can be stitched together using dedicated software such as Audacity (Audacity, Inc). However, finding available sound sources and creating an audio clip is not always easy. Furthermore, ensuring that transitions between clips are undetectable and ensuring proper fading can be challenging. Discrepancies that cannot be heard during postprocessing may be noticeable when played on higher-quality devices. This makes creating high-quality audio clips difficult and time-consuming.

Therefore, a final option for adding auditory content to the videos is the purchase of professionally recorded sounds. As with the video content, longer-duration, nonlooping content may be limited, though it is not impossible to obtain. It should be noted that the misalignment of audio and video is detectable by a trained ear, and certain viewers may be perturbed if actions such as footsteps can be seen but not heard [30].

### The Creation and Processing of the Final 360-Degree Footage

Recording the desired content using a 360-degree camera is only part of the process. The stitching and postprocessing of the footage are both equally important components of video creation and may require special consideration, as outlined below.

#### Stitching and Auto-Stitching

As 360-degree VR recordings use multiple cameras, as described above, stitching is an important part of the postprocessing step. During stitching, the videos recorded from each camera are merged into a single file, such that there is no clear start or end to the visual field as the user turns around. However, the process of merging these videos is not trivial, as the recorded videos have overlapping fields of view. This means that each lens captures a portion of the surrounding environment that is also captured by a neighboring lens. The video must, therefore, be properly overlapped to avoid double vision. This process is easily accomplished for still pictures or videos with little activity and becomes more challenging with increasing activity as objects can pass over these stitch lines.

As this process can be difficult and time-consuming, many 360-degree cameras now come with proprietary software that automatically stitches the footage together. Such software can produce relatively good results, particularly when there is limited activity or when objects and activity take place further from the camera. The recommended minimum distance that should be maintained between all activity and the camera to ensure the best result is defined in the camera’s user manual (typically 1.5 m).

Finally, if the proprietary software is unable to generate smooth stitch lines, more advanced programs such as Mistika VR (Soluciones Graficas Por Ordenador SL) can be used to improve the final video. These programs allow the stitch lines to be visualized and manually adjusted through edge points so that they do not run directly through moving objects or elements that are important to the video. Valuable written advice and video tutorials made by content creators as well as the developers of these different stitching programs are available on the web; these resources describe the steps needed to improve the stitching of a video in great detail.

#### Postprocessing

Postprocessing is an important step that allows the user to add external audio tracks, improve lighting, make color adjustments, and remove unwanted objects. Various programs can be used for postprocessing; some require the purchase of a paid license, while others are free. A detailed description of programs used by the study team can be found in the example provided in Multimedia Appendix 1.

If the camera’s built-in microphone is used, then the video and audio files will be automatically loaded into the program simultaneously. Alternatively, if the audio files are recorded using an external device, stitched together, or purchased, then they must be added to the video file separately. This can be done before or after editing the visual content, as these files are independent of each other.

Lighting and color adjustments can also be made during postprocessing. By following photographic principles, the lighting and colors in the video can be adjusted to convey a specific tone, mood, and atmosphere. The user may also wish to remove certain objects from the recorded footage, such as the camera’s tripod. Alternatively, it may be easier to cover an object rather than edit it out, for example, with a company logo. Generally, video editing programs allow users to cut out unwanted objects or color over them. If this approach is taken, then it should be noted that, depending on the duration of the video, the lighting in the scene may change. Therefore, the process of editing or removing objects may need to be done in multiple steps to ensure that the lighting and colors match. Additionally, with longer-duration videos, there is also a higher likelihood of objects in the surrounding environment interfering with the recording, such as insects landing on the camera lens. These can also be edited out during postprocessing.

In the final postprocessing step, certain settings may have to be altered to ensure that the final video is compatible with the hardware used to display it (Table 3). Within the postprocessing software itself, it may also be necessary to indicate that the content is for VR purposes and, subsequently, whether the content is monoscopic or stereoscopic. The resolution, frames per second, and necessary codecs can also be adjusted at this stage. The playback device may also require filenames to be formatted in a specific way so that the device can recognize 360-degree content. Multimedia Appendices 2 and 3 contain examples of video clips exported using the settings listed in Table 3.

**Table 3:** Video export settings for playback using a Pico G2 4K virtual reality headset (PICO).

Attribute	Setting
Codec	H.264
Width (pixels)	5760
Height (pixels)	2880
Frame rate (frames per second)	24
Aspect ratio	Square pixels
Bitrate	Variable
Virtual reality mode	Monoscopic (360° × 180°)
Time interpolation	Free sampling
Metadata	Enabled

### Hardware for Deployment and Visualization

#### Choice of Device

Once the video files have been exported, the 360-degree videos can be played on a computer, mobile phone, or VR headset. Videos on a computer or mobile device may require specific software or can be viewed directly through YouTube (YouTube, LLC) or Facebook (Meta Platforms, Inc), assuming that the video was properly uploaded for 360-degree playback on these platforms. On these devices, the virtual environment can be explored by panning around the scene. On mobile devices, the user can explore the scene by pointing the device in the direction they wish to look. This is similar to VR headsets, in which the scene rotates as the user moves their head, immersing the user in the virtual environment. In this way, exploration of the scene using VR is achieved very naturally.

Currently, there are several commercially available HMDs for displaying immersive VR content. They can all be split into 2 main categories: tethered and untethered devices. Tethered devices use a cabled connection to a powerful computer to acquire and display the VR content and often require additional equipment such as base stations and lighthouses for tracking. In contrast, untethered devices are less powerful and may require a wireless connection to transmit and receive content, but they are cable-free. This has the advantage of making the user feel less restricted in their movements and can make the device easier to use as there are no external components to consider. Another aspect that may be relevant for use in a clinical or research setting is the ability of devices to launch in what is known as kiosk mode. This mode allows the device to run a specific application when turned on, thus requiring less hands-on manipulation of the interface.

In addition to ease-of-use considerations, the internal specifications of the device need to be considered. Devices with better specifications often cost more; more expensive devices tend to offer higher image quality, resulting in a more realistic virtual environment. This is important, as it can increase the users’ sense of presence [31]. For this reason, specifications such as the resolution per eye, refresh rate, field of view, and display type all play a role in the user experience and should be considered.

In addition to the internal specifications of the device, there are also physical components to consider. To improve user enjoyment and increase immersion within the virtual environment, the HMD should be as unobtrusive as possible [31]. This means that aspects such as weight, counterbalance, padding, and adjustability, to name a few, should be considered when selecting an appropriate device. Wearing a heavy device that does not provide adequate counterbalance (usually located at the back of the user’s head) can result in discomfort on the wearer’s nose. However, while the addition of a counterbalance may alleviate pressure on the user’s nose, it may increase their discomfort when leaning their head against a support. The material and amount of padding, as well as the ability to adjust the tightness of the device, can also play a role in the user’s overall comfort. Additionally, the ability to adjust the device’s internal lenses can allow for a clearer and more focused image to be achieved.

A final aspect that should be considered is the auditory output of the device. Some devices have integrated speakers, whereas others do not. While integrated speakers may be suitable for VR gaming, they may be less desirable if the goal is to envelop the user in the virtual environment. In this case, one may choose to use external headphones (preferably noise-canceling), which can be connected to the device, typically through a cabled connection. In general, there are no special requirements to be considered in terms of compatibility between the headphones and the VR device; any commercially available headphones can be used.

#### Hygiene

**Figure 4:**
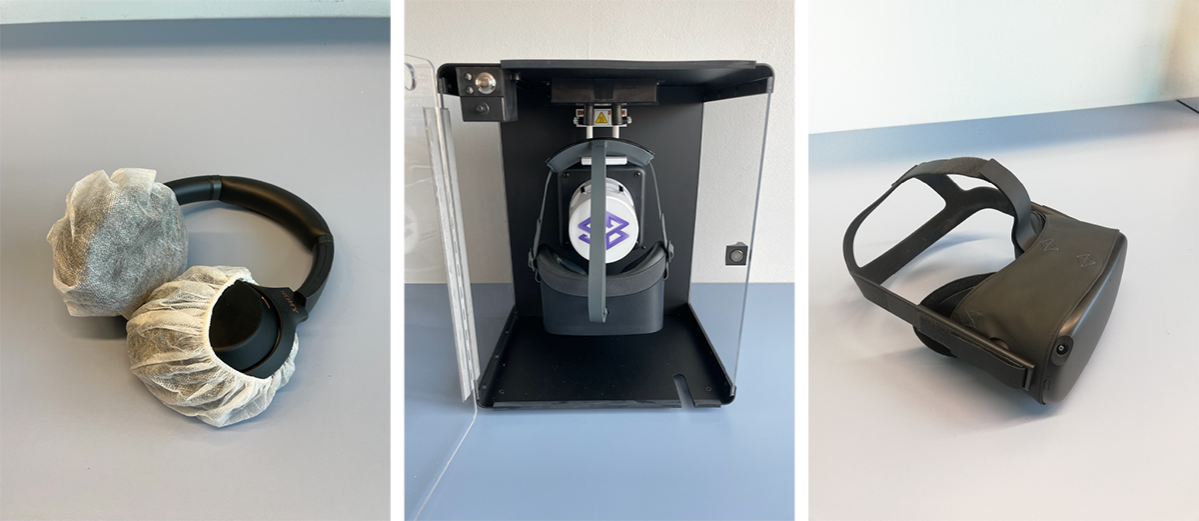
Examples of potential hygiene measures for the hardware used for deployment. (Left) Hygiene covers for over-the-ear headphones. (Middle) UVC disinfection box which can be used for head-mounted displays and headphones. (Right) Custom-made hygiene cover with fabric that can be disinfected on a first-generation Oculus Quest (Meta Platforms, Inc).

Hygiene is of particular importance when using HMDs in a clinical setting. All parts of the device must be disinfected when switching between users, and these considerations must also be accounted for when selecting a device. Devices with a plastic outer covering can be wiped clean with hospital-grade disinfectant wipes. However, some devices, such as the first-generation Oculus Quest (Meta Platforms, Inc), have a fabric covering. To overcome this limitation, a custom-made fabric cover that can be disinfected may be required (Figure 4). It should be noted that the lenses of the headset cannot be disinfected with any product that contains alcohol. For such components, a UVC disinfection box (Cleanbox Technology, Inc) can be used; this allows any VR device, including its lenses, to be safely disinfected in 60 seconds (Figure 4). Such a disinfection box can also be used to disinfect over-the-ear headphones that often contain a fabric lining inside their earpieces. Headphones can also be covered with a disposable sanitary earpiece protector to improve hygiene (Figure 4).

Arcades and specialized gaming centers are also concerned with hygiene problems but are often less strict with their requirements. These centers often use silicone covers that can be disinfected with alcohol-based wipes or disposable pads that can be placed on the portion of the headset that touches the user’s face. Although these solutions are plausible, sweating can cause silicone covers to become uncomfortable while also dampening disposable pads, causing them to slip out of place. If considering long-term use, the financial aspect of using disposable pads may also need to be considered.

### Evaluation of the VR Experience

Conducting research using VR is not limited to content; research surrounding VR also focuses on understanding the user experience, specifically as it pertains to presence and immersion within a virtual environment. There are 3 main validated questionnaires that are used to measure presence. The most cited test is a 32-item presence questionnaire developed by Witmer and Singer [32], followed by the 6-item presence questionnaire known as the Slater-Usoh-Steed questionnaire [33], and the 13-item iGroup Presence Questionnaire developed by Schubert et al [34,35]. Similarly, there are validated questionnaires concerned with immersion, such as the one developed by Tcha-Tokey et al [36], which is based on the Immersive Tendencies Questionnaire developed by Witmer and Singer [32].

In addition to measuring presence and immersion, it is important to quantify any negative side effects, known as cybersickness, caused by the virtual world [27]. These may include, but are not limited to, symptoms such as nausea, dizziness, headache, and eye strain [37]. These symptoms can be assessed by the validated Simulator Sickness Questionnaire [38]. However, care should be taken to record baseline symptoms in clinical subpopulations [37].

Finally, if the goal is to conduct a scientific study using VR, researchers may wish to consider including eye and head tracking. This could provide information about what parts of the video the user was focusing on and how much they explored their virtual environment. However, eye and head tracking are not supported by all HMDs; these requirements should be considered when selecting an appropriate HMD.

## Discussion

### Overview

Creating custom 360-degree videos for use in VR-focused research is not an easy task. Unfortunately, due to the relative novelty of the technology and its use in displaying such content, there is a lack of clear resources available, particularly for those unaccustomed to video editing. Therefore, the goal of this tutorial was to provide information pertaining to the creation, playback, and evaluation of 360-degree videos, with several concrete examples provided in Multimedia Appendices 2 and 3. This expands on existing tutorials, which provide a narrower and less complete scope of information [2,4,39].

While the use cases discussed here refer to the clinical setting, with relaxing 360-degree content provided as an example, there are also nonclinical applications for which the current tutorial could be useful [16,18,40]. As referred to throughout the tutorial, 1 nonclinical use case could involve the use of VR for guided meditation [17,18]. Another example could include using VR to explore and study architecture or improve the general well-being of individuals without access to nature [41,42]. In both of these cases, 360-degree VR content is used. Therefore, using the information provided in the tutorial, the same principles can be used to create content suited to those purposes, thereby extending the target population beyond researchers and health care professionals. In this way, this tutorial can act as a reference for anyone looking to create their own content.

### Challenges

Based on our own experiences creating 360-degree VR content, one of the greatest challenges related to creating 360-degree virtual reality content is the length of the recordings. While shorter videos that last less than 5 minutes can be recorded quite easily, there are several equipment- and environment-related issues that must be considered when longer videos are required, especially in environments that are exposed to uncontrollable factors. Not only is there an increased chance of environmental interference, such as insects landing on the lenses or individuals approaching the camera, but there are also technical challenges that must be overcome. This includes overheating and battery life, which must be considered before filming. Additionally, issues associated with file storage capacity and computational power become important when conducting postprocessing on longer videos.

Another challenge to consider when producing 360-degree videos is the content. The team associated with this study focused on recording calm scenes based on the natural environment, as the goal was to achieve a relaxation effect [13,14,41,43-45]. As such, heavy equipment often had to be brought to remote locations in a backpack. It was also difficult to find the correct balance of activity and nonactivity based on the goals of the project. For example, although the footage was intended to relax the viewer, there still needed to be enough activity to ensure that the video did not appear to be a still picture and that there was enough change in the environment to retain the viewer’s interest. Additionally, when filming in a public location, filmmakers must consider the legality of filming individuals who enter the frame of the camera. This problem is particularly relevant when recording 360-degree footage, as there is no way for an individual to avoid being filmed once they enter the camera’s field of view. One option to increase the amount of activity in a scene without running afoul of legality issues is to hire actors to create an engaging scene that is appropriate for the purposes of the content.

### Future Research

To address some of these concerns, future studies using 360-degree videos should conduct a prestudy that examines the suitability of their content for their intended purposes. In this way, the reaction of the target population can be examined at an early stage, before the investment of time and resources that are required to make all of the content. However, the suitability of the content will, to some extent, always depend on the individual. Another aspect that could be further investigated is the inclusion of different sounds. Specifically, the overall influence that the choice of sound has on the feeling of immersion within the VR environment could be examined. In this way, the user’s experience could potentially be improved.

### Conclusions

This tutorial provides users with a pipeline for the creation of customizable 360-degree videos based on first-hand experience. As the field of VR research and use continues to grow and the technology becomes more accessible to the general public, this paper will hopefully guide users through the process of creating content that is suited to their individual needs while avoiding common pitfalls associated with content creation. In doing so, this tutorial fills a gap in the literature and expands upon previously published tutorials focused on the creation of 360-degree videos by explaining 5 key considerations associated with the creation, deployment, and evaluation of 360-degree VR content.

## Appendix

Multimedia Appendix 1 Document outlining the details of the pipeline that the study team implemented while recording their nature videos.

Multimedia Appendix 2 A 2-minute, 360-degree-enabled video. The 360-degree scene can be explored by panning around the video.

Multimedia Appendix 3 A 50-second video clip of a screen capture of a 360-degree-enabled video. Exploration of the scene was done using the cursor. Note that exploration within a HMD would appear smooth. HMD: head-mounted display.

## Abbreviations

HMD: head-mounted display
VR: virtual reality
VR-CORE: Virtual Reality Clinical Outcomes Research Experts

## Data Availability

Data sharing is not applicable to this article as no data sets were generated or analyzed for this tutorial paper.
