# Supplementary material for: Creating Custom Immersive 360-Degree Videos for Use in Clinical and Nonclinical Settings: Tutorial
Source: JMIR Med Educ. 2023 Sep 14;9:e42154. doi: 10.2196/42154 (PMC10540026; doi:10.2196/42154)
Supplement: Multimedia Appendix 1 [file mededu_v9i1e42154_app1.docx]

**Appendix A**

*Example process to obtain final 360-degree footage*

Planning and Designing

While planning and designing there were many considerations that the study team kept in mind. Below we briefly list the considerations which were important for the final goal of the content, namely, to investigate the relaxing effect of VR stimulation in critically ill patients. The videos had the requirement that they needed to last at minimum 30 minutes.

- Nature based scenes with limited human activity, but still with something happening (eg, wave lapping at beach, sunset overlooking a lake, animals in a petting zoo, etc).
- Locations not too remote so that all equipment could be brought on foot by a single individual.
- Somewhere that a 30-minute recording could be done without interruption (eg, individuals coming and touching the camera, cars driving past, etc).
- For the research study it was important for our purposes that we record the same location three times, (once in the morning, once midday, and once in the evening) as we wanted to mimic the passing of time via changes in the lighting. Therefore, the location needed to be suitable in terms of activity and human presence at all three time-points.
- Areas needed to have limited human presence due to the filming laws. Where we filmed, it is allowed to film a general location, which may have a person enter the frame, but it is not allowed to film specifically an individual without their consent. However, even knowing this there were incidents where people did not like being filmed and asked for the video to be deleted. In cases where children entered the frame, attempts were made to blur their faces in the final footage.
- Not near a road or other sources of constant or loud sounds, so that on-location auditory recordings could be done.
- Nothing which could scare or be uncomfortable for our target population. This is elaborated on by Naef et al., 2022.
- Could not film in the rain.

Equipment and Recording

The devices used to record our content were as follows:

*Camera:*

- Insta360 Pro II (Arashi Vision Inc.) with a total of three battery packs. One 30-minute recording used up most of one battery, and since charging the batteries on-location was not an option, we needed to have enough to do three recordings per day. The main direction is that which one would see while standing behind the control panel and looking over the camera.
-
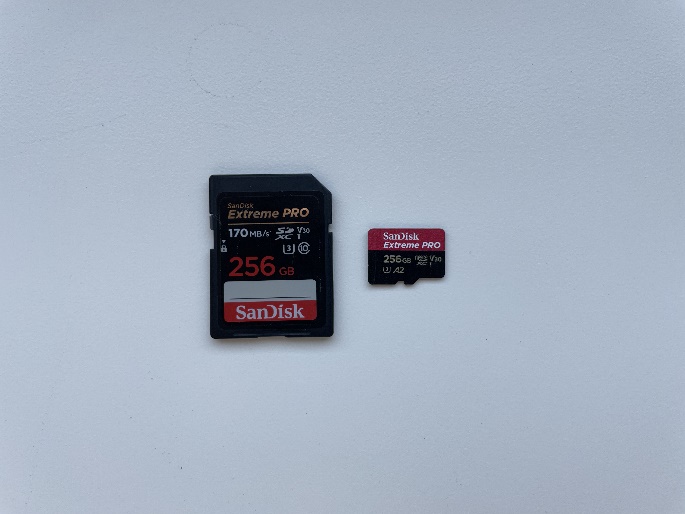
Six, 256 GB micro-SD cards, that is one for each lens. A 30-minute recording (6k|60fps|3D), per lens, is roughly 27 GB in size, that is 163 GB for all six lenses which need to be stitched together.
- One, 256 GB SD card, that is one main storage for the device. A 30-minute recording (6k|60fps|3D) results in roughly 7.4 GB of data on the main SD card when no audio is recorded.

*Audio:*

-
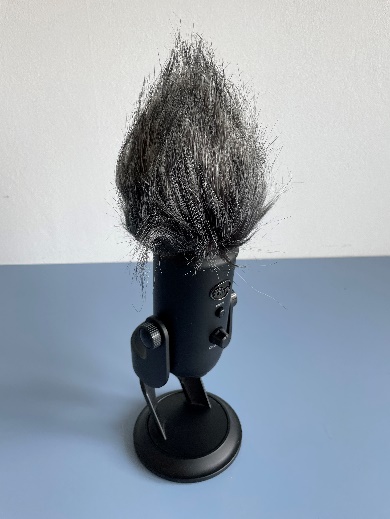
Internal Insta360 Pro II(Arashi Vision Inc.) microphone. This was not used as the goal was to record for 30 minutes, and the built-in fan turns on automatically after 10 minutes.
- Blue Yeti Blackout USB Microphone with universal windscreen. Stereoscopic sound was recorded but the sounds were too strong. Distant roads and sounds not audible during the actual recording were picked up by the microphone, therefore, these sounds were not used.
- Existing nature soundscape recordings were purchased from Listening Earth. This is one of the few companies that provides long duration (>30-minutes) non-looping nature soundscapes.

*Computer:*

- CPU: AMD Ryzen Threadripper 3970X, 32 Core
- GPU: nVidia RTX Titan (24GB)
- RAM/Memory: AsRock Creator TRX40, 256GB RAM
- SSD: Samsung 970 EVO Plus, 2TB
- HDD: 4x Seagate IronWolf (8TB, 3.5”, CMR)

*Miscellaneous:*

- Cleanbox CX1, UVC hygienic system (Cleanbox Technology Inc)
- Disinfectant wipes
- Headphone Covers

Creating and Processing

Before describing the exact pipeline, we used in creating our footage, the study team would like to make clear that there are many ways to achieve the end goal. We hope that this can act as a guideline to possible steps which may be needed to achieve your desired results. There are many different programs and methods which can be used, this is just an example of one such process.

To create our content, we opted to purchase licenses for both Mistika VR (Soluciones Graficas Por Ordenador SL) and Adobe Premiere Pro (Adobe Inc.). While similar results could have surely been achieved using non-licensed programs, we felt that the additional documentation and tutorials that come with paid programs was worth the cost.

*Setup*:

- Move files from the SD cards to the HDD. Only keep files related to the video actively. being edited on the SSD to ensure enough space for all the raw and intermediary files.

*Stitching*:

- Load all six video files, one per lens, into the Insta360 Stitcher program (Arashi Vision Inc.).
- Select a reference frame and open the *preview stitching effect* window.
- Turn on Zenith Optimization and click *save and apply* when it’s complete. This creates a *.log* file containing the stitch information and is stored in the *log* folder associated with the program.
- Open Mistika VR (Soluciones Graficas Por Ordenador SL) and create a new project with the video format and frame rate matching your files.
- Load the six video files, one per lens, into Mistika VR (Soluciones Graficas Por Ordenador SL).
- Import the *.log* file created by the Insta360 Stitcher program (Arashi Vision Inc.).
- If desired, you can change the main direction of the videos at this point by dragging the scene. Set the main area of interest to be in the middle. Be careful not to change the horizontal line too much.
- Improve the offsets and angles of the stitching, available under the positions tab in the left-side menu.
- Complete horizon alignment.
- Select optical flow and change the feather to 30.
- Verify the placement of the edge points and ensure that no important object coincides directly with the stitch line.

*Export Stitched Video*

- Export the stitched video from Mistika VR (Soluciones Graficas Por Ordenador SL) as ProRes to retain as much quality as possible for further editing.
- Export first a short section to ensure that all settings are as desired.
- Export full content if all settings are as desired. Longer videos may need to be exported in smaller sections to avoid corruption (eg, separate a 30 minute video into two 15 minute exports).

| Attribute | Setting |
| --- | --- |
| File Type | Quicktime ProRes |
| Codec | Mov ProRes |
| Stereo | No Stereo |
| Audio | None (as we added the audio file later) |

*Further Editing:*

- Import the ProRes file created by MistikaVR (Soluciones Graficas Por Ordenador SL) into Adobe Premiere Pro (Adobe Inc.).
- Complete and color corrections, object removal, fading, addition of text, etc. at this stage.
- Add the audio file by importing it to the same Adobe Premiere Pro (Adobe Inc.) project.
- Complete any fading or edits desired on the audio file at this stage.
- Verify that the sequence settings for the project are correct (eg, frame size, frames per second, etc.), and that the content is recognized as being VR. If correctly identified, the sequence settings should have a VR section.


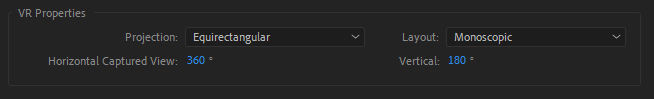


- Export the final video from Adobe Premiere Pro (Adobe Inc.). The settings we used can be seen below. Settings not listed here were left as set by default.
- The final size for a 30 minute, 5K, 24 fps video, was roughly 1GB.

| Attribute | Setting |
| --- | --- |
| Codec | H.264 |
| Preset | Adaptive High Bitrate |
| Width x Height | 5760 x 2880 |
| Frame Rate | 24 |
| Aspect | Square Pixels |
| Bitrate | VBR, 1 Pass |
| Virtual Reality Mode | Monoscopic (360°x180°) |

Deployment and Visualization

The videos created were first tested on a first-generation Oculus Quest. At that time, to achieve no dropped frames the following export settings were required.

| H264 | H265 |
| --- | --- |
| ~ 4864 x 2432, 60 fps | ~ 4480 x 2240, 60 fps |
| ~ 3400 x 3400, 60 fps | ~ 3168 x 3168, 60 fps |

Filenames also had to be as follows:

*filename*_360.mp4 for monoscopic

*filename*_360_TB.mp4 for 360 top/bottom stereoscopic

*filename*_180_3D.mp4 for 180-degree side-by-side or top/bottom

However, due to feedback from our participants regarding weight and comfort of this headset, as well as other requirements, such as the Kiosk mode which was not yet enabled on the Oculus Quest, we decided to proceed with the Pico G2 4k for future studies. The Pico G2 4k had the benefit not only of allowing us to run the headset in kiosk mode, but also to call our videos from a Unity program to allow for easy calibration once the user was wearing the device. This was important as participants in our study were lying down, therefore, to avoid the pitfall that they only see the sky of the virtual environment, the video itself had to be shifted appropriately. In terms of video encoding formats for the Pico G2 4K there are a few different options. From their official website (https://sdk.picovr.com/docs/FAQ/chapter_four.html) you will see the following recommended and maximum values. However, we chose to lower the frames per second (FPS) as we felt this helped decrease, or make less noticeable, the flickering in the brighter videos.

| Parameter | Recommended | | Maximum | | Implemented |
| --- | --- | --- | --- | --- | --- |
| Resolution | 3840*2160 | 3840*3840 | 4096*4096 | 5760*2880 | 5760*2880 |
| FPS | 60 | 30 | 30 | 30 | 24 |
| Bitrate (Mbps) | 40 | 50 | 100 | 100 | Variable |
| Decoder Standard | H.264, H.265 | H.264, H.265 | H.265 | H.264 | H.264 |

Evaluation of Experience

In our experiment, for which we used these videos, we were not focused on measuring the level of presence and immersion, therefore, we did not use a validated questionnaire. Instead, questions we found relevant to our study question were extracted from validated questions. Additional questions such as “How aware were you of people in your surrounding environment during the VR stimulation?” were added to our questionnaires as they were relevant to our study question.

Cybersickness questions were included and asked immediately following the VR and again 30-minutes post VR.
